# Supplementary material for: Specific ablation of the NCoR corepressor δ splice variant reveals alternative RNA splicing as a key regulator of hepatic metabolism
Source: PLoS One. 2020 Oct 26;15(10):e0241238. doi: 10.1371/journal.pone.0241238 (PMC7588069; doi:10.1371/journal.pone.0241238)
Supplement: S1 File — (PDF) [file pone.0241238.s001.pdf]

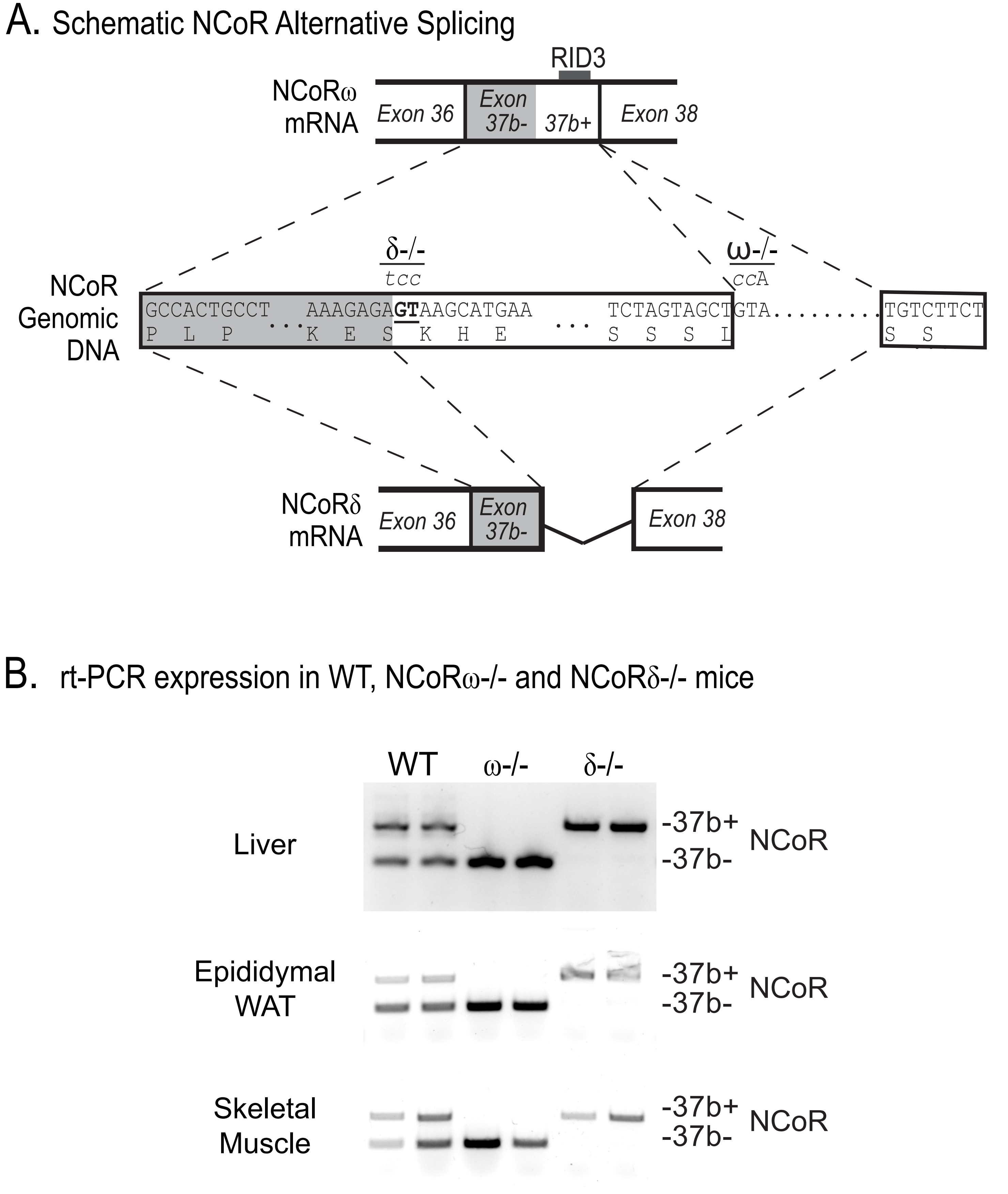

Supplemental Figure S1
Goodson et al.

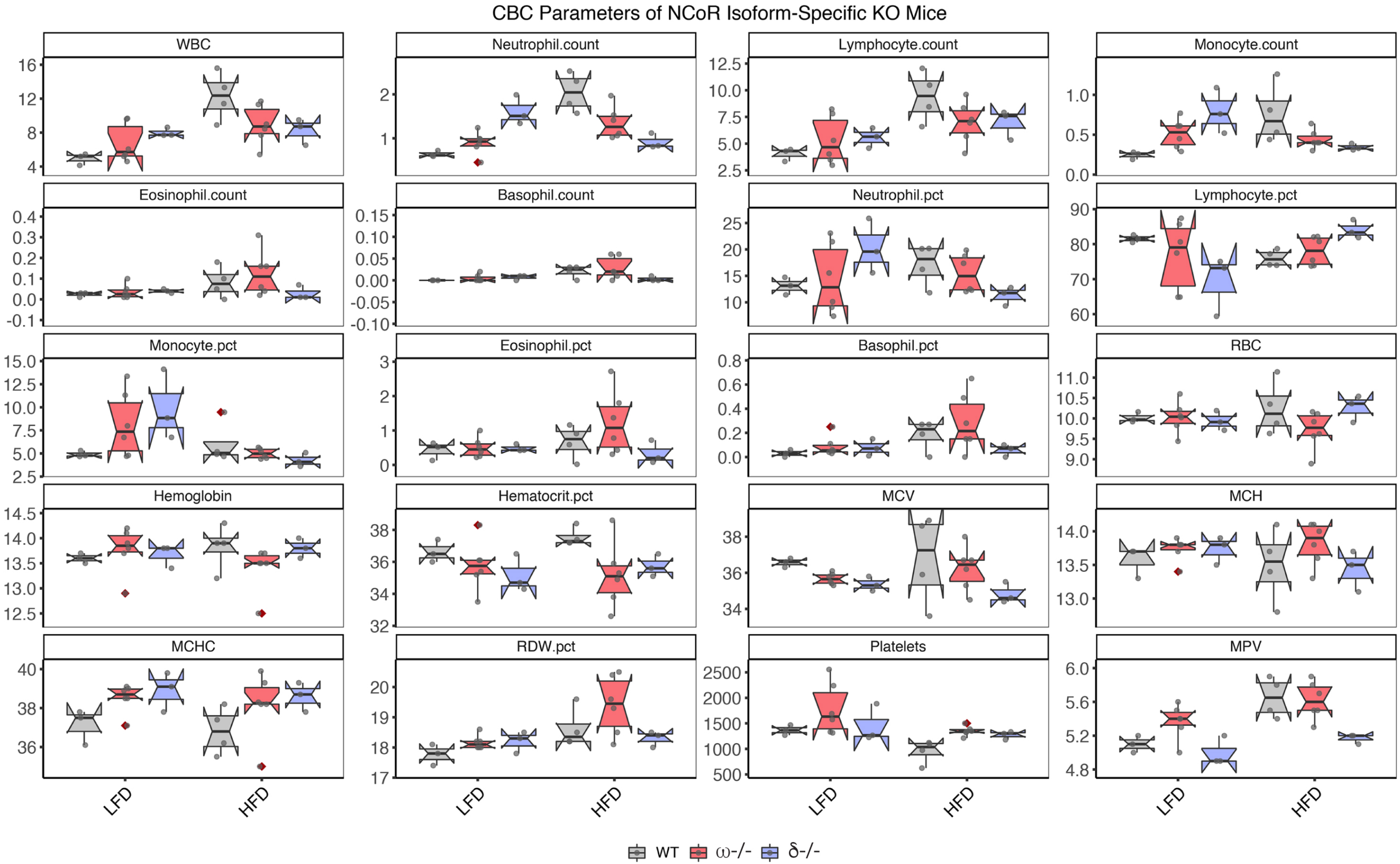

Supplemental Figure S2
Goodson et al.

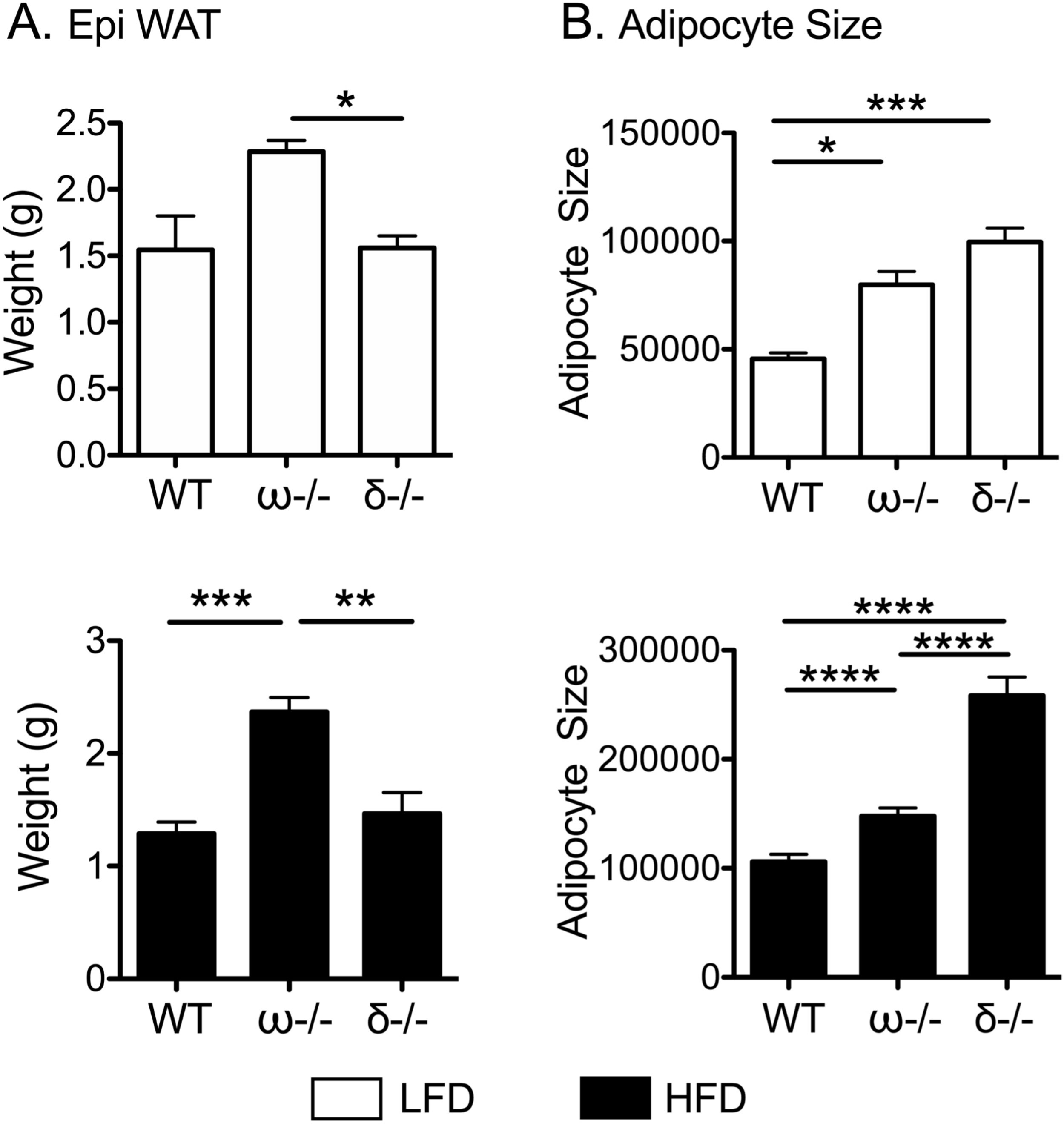

Supplemental Figure S3
Goodson et al.

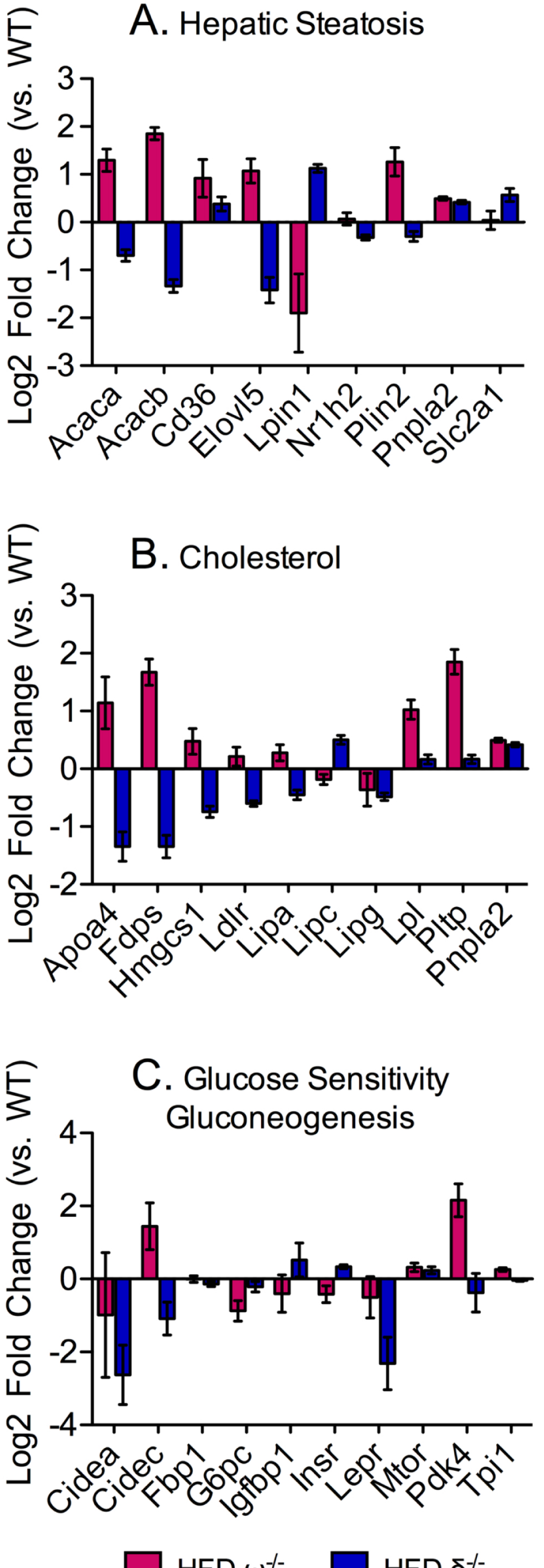

Supplemental Figure S4
Goodson et al.

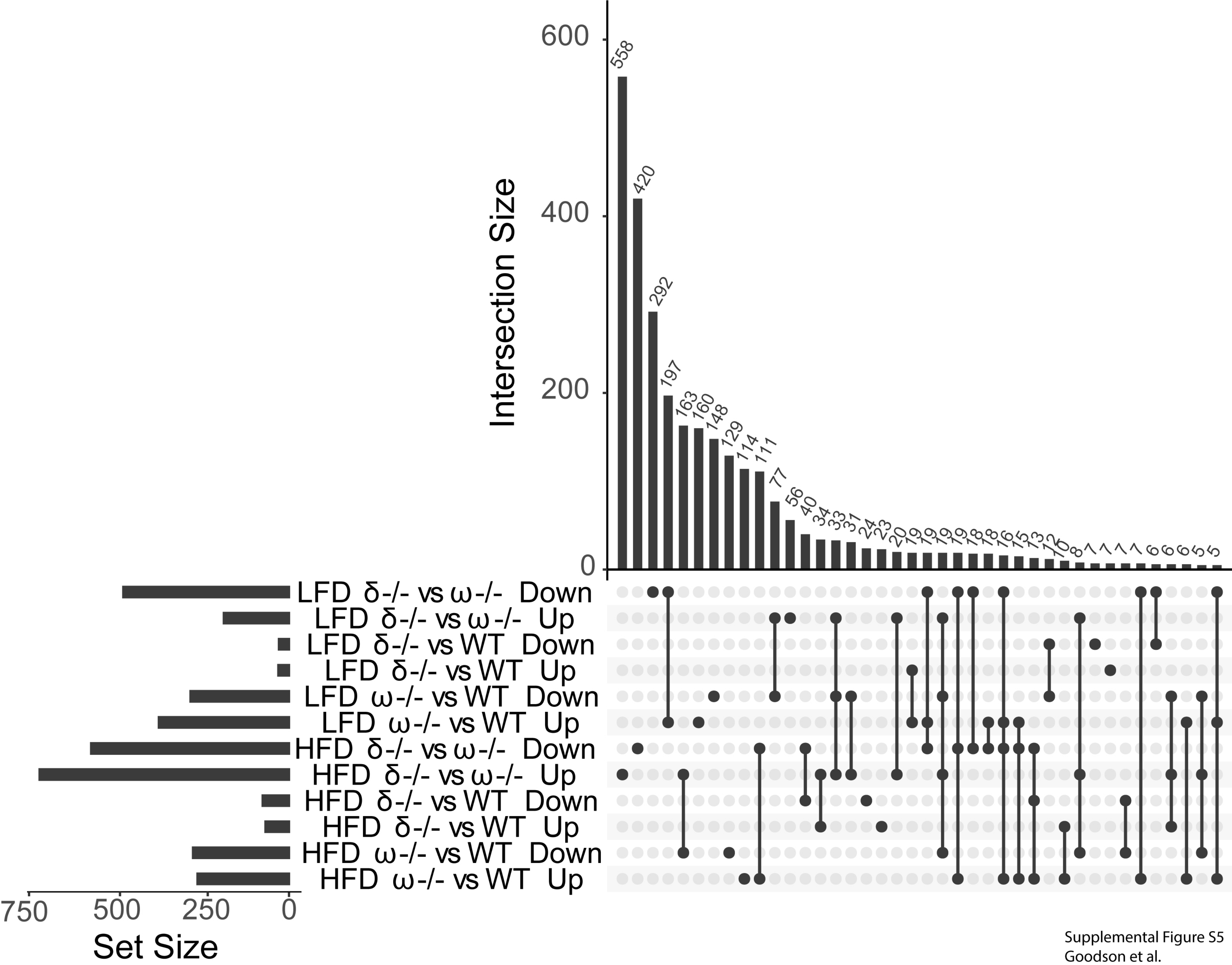

Supplemental Figure S5
Goodson et al.

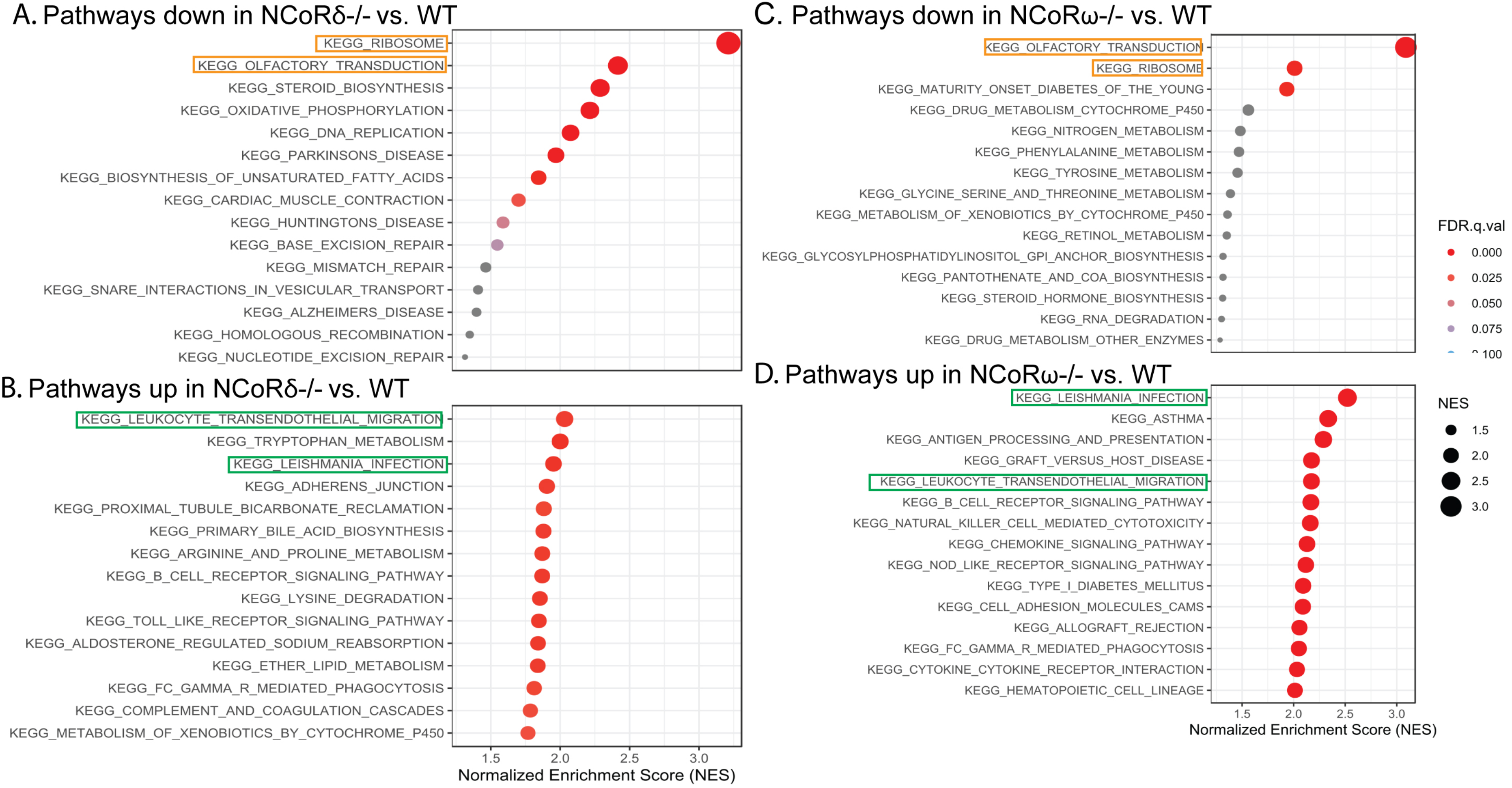
A. Pathways down in NCoRδ-/- vs. WT
KEGG_RIBOSOME
KEGG_OLFACTORY_TRANSDUCTION
KEGG_STEROID_BIOSYNTHESIS
KEGG_OXIDATIVE_PHOSPHORYLATION
KEGG_DNA_REPLICATION
KEGG_PARKINSONS_DISEASE
KEGG_BIOSYNTHESIS_OF_UNSATURATED_FATTY_ACIDS
KEGG_CARDIAC_MUSCLE_CONTRACTION
KEGG_HUNTINGTONS_DISEASE
KEGG_BASE_EXCISION_REPAIR
KEGG_MISMATCH_REPAIR
KEGG_SNARE_INTERACTIONS_IN_VESICULAR_TRANSPORT
KEGG_ALZHEIMERS_DISEASE
KEGG_HOMOLOGOUS_RECOMBINATION
KEGG_NUCLEOTIDE_EXCISION_REPAIR
B. Pathways up in NCoRδ-/- vs. WT
KEGG_LEUKOCYTE_TRANSENDOTHELIAL_MIGRATION
KEGG_TRYPTOPHAN_METABOLISM
KEGG_LEISHMANIA_INFECTION
KEGG_ADHERENS_JUNCTION
KEGG_PROXIMAL_TUBULE_BICARBONATE_RECLAMATION
KEGG_PRIMARY_BILE_ACID_BIOSYNTHESIS
KEGG_ARGININE_AND_PROLINE_METABOLISM
KEGG_B_CELL_RECEPTOR_SIGNALING_PATHWAY
KEGG_LYSINE_DEGRADATION
KEGG_TOLL_LIKE_RECEPTOR_SIGNALING_PATHWAY
KEGG_ALDOSTERONE_REGULATED_SODIUM_REABSORPTION
KEGG_ETHER_LIPID_METABOLISM
KEGG_FC_GAMMA_R_MEDIATED_PHAGOCYTOSIS
KEGG_COMPLEMENT_AND_COAGULATION_CASCADES
KEGG_METABOLISM_OF_XENOBIOTICS_BY_CYTOCHROME_P450
1.5
2.0
2.5
3.0
Normalized Enrichment Score (NES)
C. Pathways down in NCoRω-/- vs. WT
KEGG_OLFACTORY_TRANSDUCTION
KEGG_RIBOSOME
KEGG_MATURITY_ONSET_DIABETES_OF_THE_YOUNG
KEGG_DRUG_METABOLISM_CYTOCHROME_P450
KEGG_NITROGEN_METABOLISM
KEGG_PHENYLALANINE_METABOLISM
KEGG_TYROSINE_METABOLISM
KEGG_GLYCINE_SERINE_AND_THREONINE_METABOLISM
KEGG_METABOLISM_OF_XENOBIOTICS_BY_CYTOCHROME_P450
KEGG_RETINOL_METABOLISM
KEGG_GLYCOSYLPHOSPHATIDYLINOSITOL_GPI_ANCHOR_BIOSYNTHESIS
KEGG_PANTOTHENATE_AND_COA_BIOSYNTHESIS
KEGG_STEROID_HORMONE_BIOSYNTHESIS
KEGG_RNA_DEGRADATION
KEGG_DRUG_METABOLISM_OTHER_ENZYMES
FDR.q.val
0.000
0.025
0.050
0.075
D. Pathways up in NCoRω-/- vs. WT
KEGG_LEISHMANIA_INFECTION
KEGG_ASTHMA
KEGG_ANTIGEN_PROCESSING_AND_PRESENTATION
KEGG_GRAFT_VERSUS_HOST_DISEASE
KEGG_LEUKOCYTE_TRANSENDOTHELIAL_MIGRATION
KEGG_B_CELL_RECEPTOR_SIGNALING_PATHWAY
KEGG_NATURAL_KILLER_CELL_MEDIATED_CYTOTOXICITY
KEGG_CHEMOKINE_SIGNALING_PATHWAY
KEGG_NOD_LIKE_RECEPTOR_SIGNALING_PATHWAY
KEGG_TYPE_I_DIABETES_MELLITUS
KEGG_CELL_ADHESION_MOLECULES_CAMS
KEGG_ALLOGRAFT_REJECTION
KEGG_FC_GAMMA_R_MEDIATED_PHAGOCYTOSIS
KEGG_CYTOKINE_CYTOKINE_RECEPTOR_INTERACTION
KEGG_HEMATOPOIETIC_CELL_LINEAGE
NES
1.5
2.0
2.5
3.0
Normalized Enrichment Score (NES)

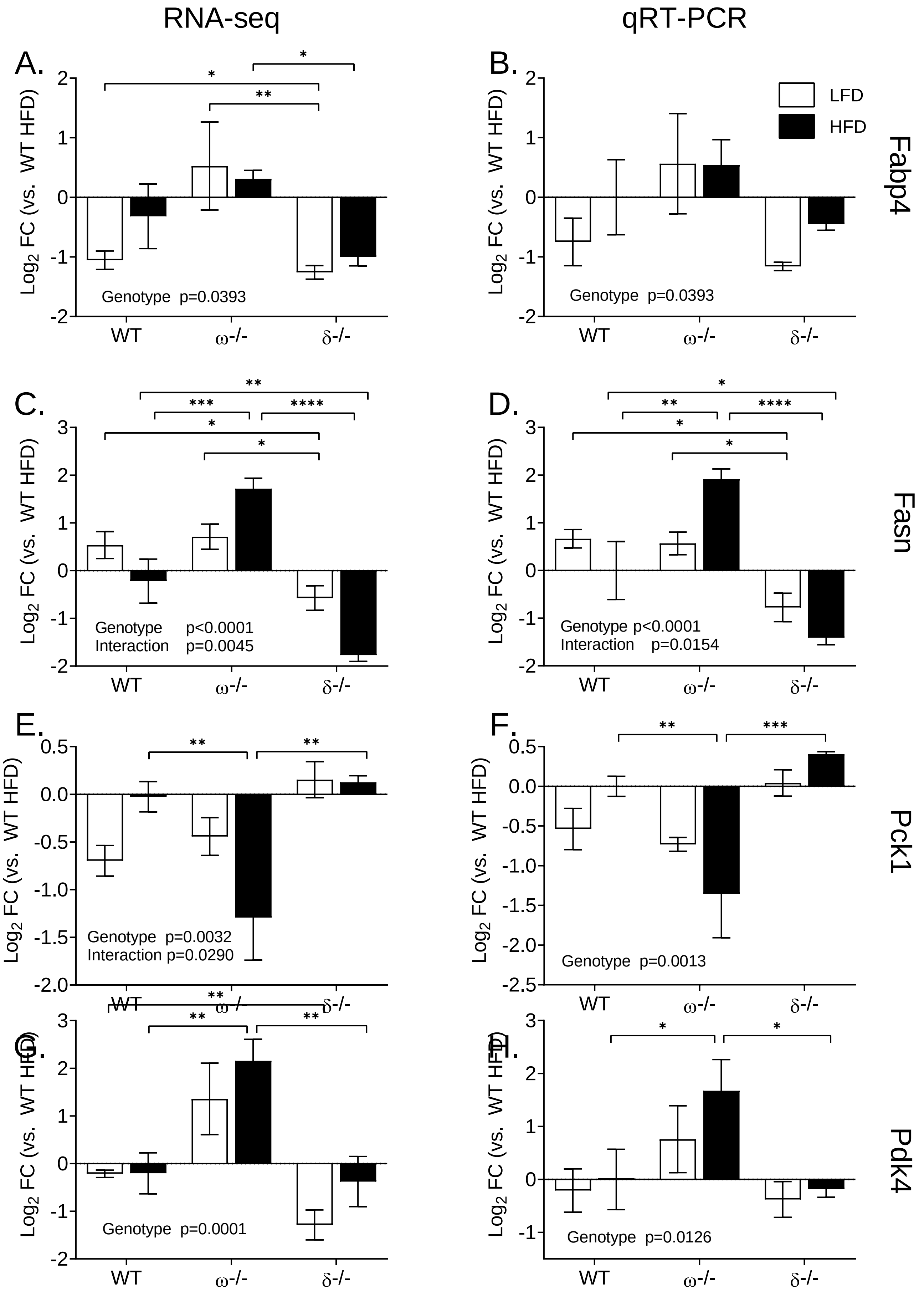

Supplemental Figure S7
Goodson et al.

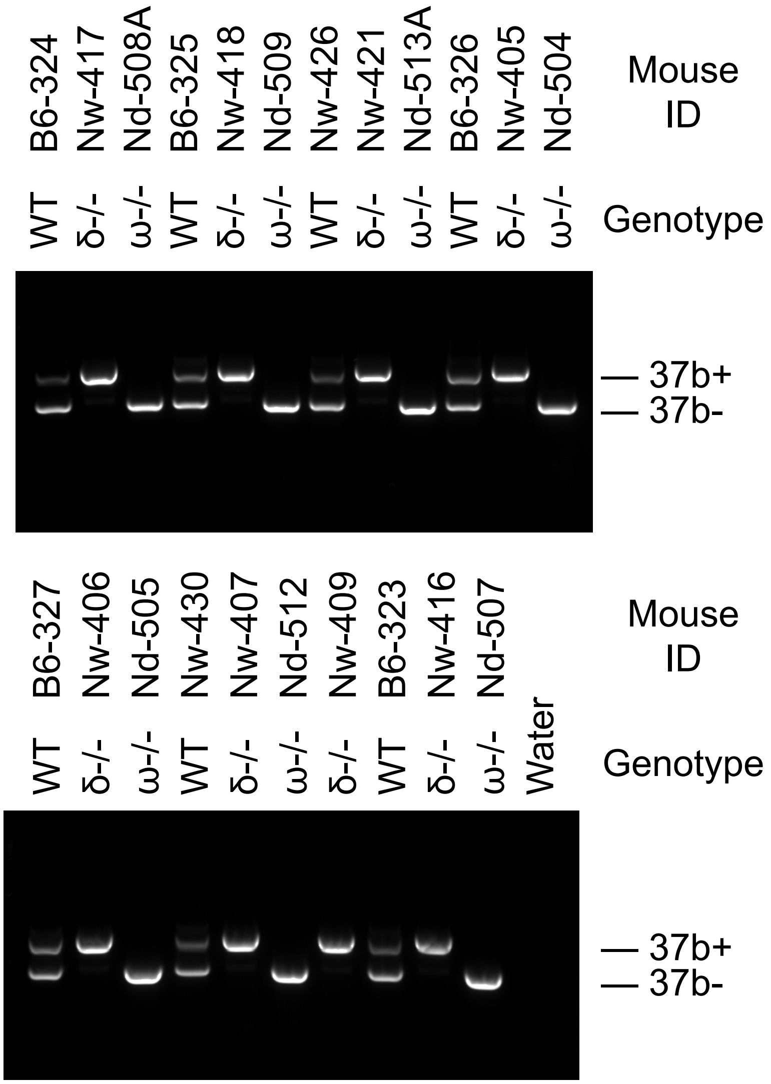

Supplemental Figure S8
Goodson et al.
