# Supplementary figures and images for: Specific ablation of the NCoR corepressor δ splice variant reveals alternative RNA splicing as a key regulator of hepatic metabolism
Source: PLoS One. 2020 Oct 26;15(10):e0241238. doi: 10.1371/journal.pone.0241238 (PMC7588069; doi:10.1371/journal.pone.0241238)

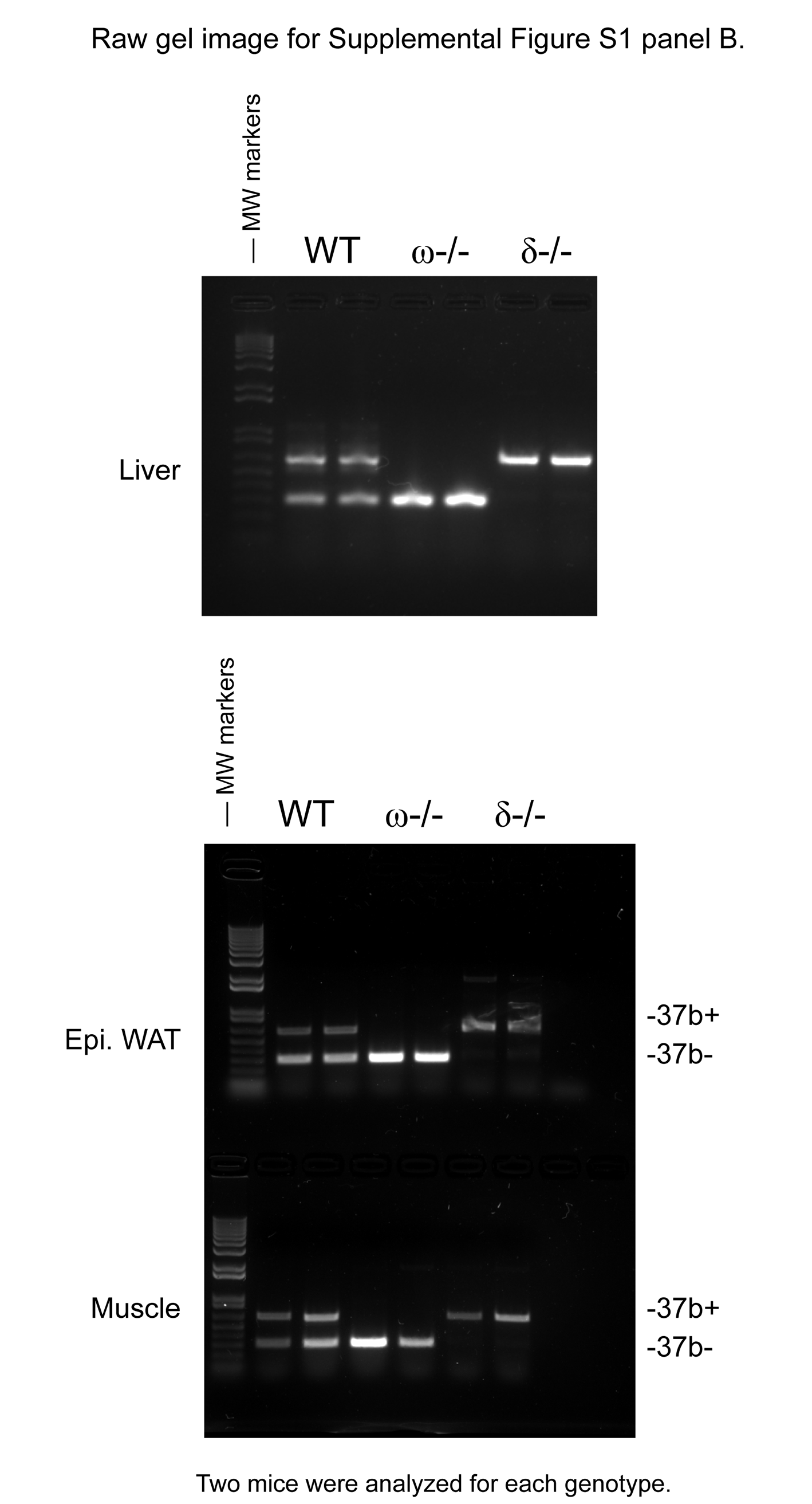

Supplement: S1 Raw image — (TIF) [file pone.0241238.s005.tif]
