# Supplementary material for: Specific ablation of the NCoR corepressor δ splice variant reveals alternative RNA splicing as a key regulator of hepatic metabolism
Source: PLoS One. 2020 Oct 26;15(10):e0241238. doi: 10.1371/journal.pone.0241238 (PMC7588069; doi:10.1371/journal.pone.0241238)
Supplement: Attachment — Submitted filename: Authors response 091720.docx [file pone.0241238.s006.docx]

Authors' response to reviewers' comments on PONE-D-20-17204 (Marty's 2nd draft):

We sincerely thank the reviewers and the editor for their efforts on our behalf. We believe that their suggestions and insights have significantly improved our study. We hope that we have adequately answered their concerns and that the revised manuscript is now acceptable for publication. We detail our response below:

**Editorial advice: PLOS ONE does not permit use of "data not shown."**

We have now either provided the data or, if the original observation was non-essential, we eliminated it from the revised manuscript.

In addition, we have deposited the results of the RNAseq analysis from this paper in the NCBI’s Gene Expression Omnibus and included the GEO Accession number (GSE157761; lines 168-169).

**Reviewer #1 considered our manuscript to be an interesting article but expressed some notable concerns, specifically:**

1. NCoR contains three C-terminal RIDs, NCoR only two, and here, the differential gene expression profiling between NCoR-/- and NCoR-/- mice had been identified by RNAseq, so, NCoR, as a nuclear Co-Repressor, could interact with different nuclear receptors and non-receptor transcriptional factors to repress gene expression. Please test the differential partner factors profiling between NCoR and NCoR by IP associated with MS.

We agree with the reviewer as to the value of defining the different partner interactions mediated by the two different corepressor splice variants. In response we have now provided the results of a series of GST-pulldown experiments. We employed the GST-pulldown approach in place of the IP/MS method for several reasons. Due to financial limitations we do not have any live colonies of our NCoR splice-specific mice. It would therefore require many months (and resources we do not have) to recover homozygotes from our frozen sperm samples as the first step to perform these experiments. More so, in our hands we have found the IP/LC/MS/MS approach itself requires substantial time and resources to perform (as well as representing a somewhat "fussy" method to quantitate reliably). We hope the reviewer will accept the GST-pulldowns, provided as data within the revised text (lines 291 to 295), to be an acceptable substitute.

2. Please confirm the results of RNAseq by qPCR, such as FASN, PDK4, FABP4, PCK1, etc.

This information is now provided as the new Supplemental Fig. S7 (also please see line 371).

**Reviewer #2 viewed the original as addressing an important topic relevant for the better understanding of transcriptional networks governing liver and whole-organism physiology. Although viewing it a merit to take on this "challenging task" reviewer #2 felt the study as originally submitted had flaws in its controls and mechanistic approaches. Specific concerns were:**

(1) The knockout mice are insufficiently characterized. The relative expression levels (mRNA, protein) of the NCoR WT, delta versus omega splice variants in WT and KO livers, and other tissues including adipose tissue, are not documented. The reader does not know which tissues are affected and what is the ratio between WT and splice variant. It is insufficient to refer to previous papers, the expression in the mice used for the current studies must be reported.

We have now provided RT-PCR expression data for liver and a variety of additional tissues. In summary, in all tissues examined NCoR𝛿 mRNA expression was undetectable in the NCoR𝛿-/- knockout and NCoR𝜔 mRNA expression was undetectable in the NCoR𝜔-/- knockout (revised Supplemental Fig. S1B and line 198). We also confirmed the NCoR splice-variant mRNA levels from each of the liver samples used for the RNAseq analysis in Supplemental Fig. S8.

We would have preferred to also provide protein expression levels. Unfortunately the NCoR𝛿 versus NCoR𝜔 proteins can not be distinguished by electrophoresis/immunoblot. The problem arises from these splice variants migrating at essentially indistinguishable electrophoretic mobilities (due to their very similar large sizes, both over 240,000 kDa), and from the inability to generate NCoR𝛿-specific antibodies (NCoR𝛿 differs from NCoR𝜔 due only to the loss of exon 37b+ sequencces; as a consequence there are no NCoR𝛿 unique epitopes to which to raise antisera specific to this variant). Given the undetectable levels of NCoR𝛿 and NCoR𝜔 mRNA in the respective knockouts, however, it is very unlikely that there is any significant corresponding protein expression in these animals. This is discussed in the revised text (lines 201-205). It should also be noted that these mice were generated to contain a germline (global) mutation that ablates the requried GT nucleotide sequence of the 5’ splice donor site for either the NCoR𝛿 or the NCoR𝜔 splice-variant, rather than by using *in vivo* flox methodologies. Homozygosity each mutation was confirmed by genotyping in all mice. Conceptually NCoR𝛿-/- and NCoR𝜔-/- mice should retain no residual WT NCoR loci in any tissue.

(2) The mouse experiments are statistically not sound, since the numbers of mice for each experiment are not indicated. This applies for RNA-seq as well, how many biological replicates were sequenced? It is even not indicated whether male or female mice were used.

We now indicate that all the mice analyzed were males (lines 127, 539-541) and provide "n" values for our experiments in the revised Figure Legends.

(3) The interpretations of the diverse phenotypes are weak, e.g. direct effects of NCoR function in the liver (hepatocytes) are not distinguished from tissue crosstalk effects, for example adipose tissue (JBC2011) to the liver. Experiments using isolated hepatocytes from WT versus KO mice would help to distinguish these alternatives.

### Unfortunately we were forced to terminate our colonies of NCoR splice-specific knockout mice and it would take many months (and more resources than currently available to us) to recover homozygous animals from frozen sperm and to perform the hepatocyte experiment requested. We fully recognize that tissue autonomy is a valuable and interesting question but respectfully put forward that it was not the core question we were seeking to address in these, our first-step characterizations of the NCoR𝛿-/- animals. Our goal at this stage was to determine the overall NCoR𝛿-/- phenotype at the organismal level; we hope to have an opportunity to track down the specific tissue origins of each of the phenotypes in future follow-up experiments. Although a "*Tu Quoque*" defense, similar first reports of gene KOs have similarly often postponed analysis of autonomous versus non-autonomous tissue effects to later study. We fully appreciate the reviewer's point, however, and we now discuss this issue in the revised manuscript (lines 433 to 439).

(4) Major conclusions must be derived from comparisons of WT vs NCoR delta KO and WT versus NCoR omega KO, not from comparing delta versus omega. Specifically, there seems no statistical difference between WT and NCoR delta KO mice on TG and liver weight increase upon HDF. As an example, the authors find ‘that NCoR delta -/- mice exhibited greatly reduced hepatic steatosis as well as reduced overall weight gain on an HFD compared to the NCoR omega -/- mice. The NCoR delta -/- mice also fail to demonstrate the strong resistance to diet-induced glucose intolerance exhibited by the NCoR omega-/- animals.’ If they would have compared NCoR delta -/- mice to WT mice, the alternative interpretation would have been that the delta splicing variant has only moderate or no major functions in the liver.

Our primary goal in this manuscript was to examine and contrast the splice-specific effects of NCoR𝛿 and NCoR𝜔; we therefore found it useful to present our narrative more in terms of the similarities and differences between the NCoR𝛿-/- and NCoR𝜔-/- mice than in terms of comparisons of the mutants to WT. Nonetheless we do also present and discuss all of the corresponding mutant to WT comparisons for the benefit of readers seeking to examine this work from that perspective (e.g. lines 90-93, 207, 212-214, 230-235, 252-255, 272-276, 388-393, 404 to 405, 407 to 412, 429 to 432).

We also note that although the effects of the NCoR𝛿-/- KO are generally modest compared to WT, the NCoR𝛿-/- KO nonetheless presents with a number of significant phenotypic differences relative to WT, including alterations in hematopoiesis, distinct body weight gains on low and high fat diets, and differences in liver gene expression profiles. Further, given NCoR𝛿 and NCoR𝜔 are nearly equally expressed in WT liver and in many other tissues, determining the actions of both variants represents a key requirement for fully understanding the role of alternative NCoR splicing. The unexpectedly relatively modest hepatic effects of ablating NCoR𝛿, compared to the more dramatic effects of ablating NCoR𝜔, was therefore important to establish. We have included and expanded our discussions of these issues in the revised manuscript (lines 207-209, 230-231, 252 to 258, 384 to 391, 404 to 406, 429 to 432).

(5) Fig. 3 A, B: Why does HFD feeding not effect liver weight and liver triglycerides (TG) in WT mice? This questions whether the mice respond appropriately. Again, there is no significant difference between WT and delta KO mice in weight and TG, so no liver phenotype of this splice variant.

We are unclear as to why we fail to see a more robust difference in liver weight and triglyceride levels between low fat and high fat diet-fed WT mice. There is a trend (p=0.079) toward increased liver weight with the HFD diet, but not a statistically robust one. This same phenomenon was also observed for WT animals in our previous NCoR𝜔-/- study (representing a completely independent feeding trial) (35). We do note that there was a clear difference in hepatic steatosis between the low and high fat diets in the NCoR𝜔-/- and NCoR𝛿-/- animals. We suspect that the extended time course (25 weeks) of our experiment and the formulation of the low fat control diet (Research Diets cat# D12450B; 35% kcal sucrose) available at the time of this study may be responsible for muting the HFD enhancement of steatosis anticipated for WT animals by increasing the steatosis in the LFD control mice. For example, it has been reported that chronic sucrose consumption in rats leads to increased hepatic steatosis (Souza Cruz EM et al., Long-term sucrose solution consumption causes metabolic alterations and affects hepatic oxidative stress in Wistar rats. Biol Open. 2020 Feb 28;9(3):bio047282. doi: 10.1242/bio.047282).

(6) Fig. 4A, C, the omega -/-mice show clearly better glucose clearance than WT mice under both LFD and HFD, while there is no difference between omega -/-and WT mice in ITT test. How could omega -/- mice have no distinguishable insulin sensitivity from WT (line 232-233), but still can greatly enhance resistance to glucose intolerance (line 227-229)? In addition, how many mice were used in each group since some SD/SE are missing. Is there data significance at any timepoints between WT and omega -/-or delta -/-?

We observed this same phenomenon previously and proposed that the unanticipated enhanced glucose tolerance of the NCoR𝜔-/- mice (despite their elevated weights and adiposity) in the absence of altered insulin signaling was due in part to a shift in hepatic gene expression away from gluconeogenesis toward lipogenesis (35). Given the HFD fed NCoR𝛿 knockout mice exhibited a “trend” toward a modestly improved ITT whereas the NCoR𝜔-/- animals did not, these observations suggest that although both NCoR splice variants may enhance glucose tolerance they do so to different extents and likely by different mechanisms. We discuss these issues in the revised manuscript (lines 514-523).

We also now provide the "n" values requested in the figure legend. None of the individual time points in Figure 4B display statistic differences between the different genotypes; the integrated area above the curve (Figure 4D) proved the best means of visualizing and analyzing this data statistically.

(7) Suggested is further to discuss better what we already know about the function of NCoR in the liver, without and within the entire corepressor complex (e.g. as cofactor for HDAC3, driving potentially many phenotypes), and which are its most likely nuclear receptor/transcription factor targets in the liver linked to the phenotypes (and target genes) of the splice variant knockouts. For example, how much do NCoR WT and splice variant KO overlap/oppose the TR beta and PPAR alpha KO changes in the liver?

We thank the review for this suggestion and provide an expanded discussion of these points in the revised manuscript (lines 484-498).

(8) The writing accuracy has to be improved, there are errors in spelling and nomenclature. For example, in the Introduction the full names should be correctly for NCoR (nuclear receptor corepressor) and SMRT (silencing mediator of retinoic acid and thyroid hormone receptor). Suggested is also to include the official gene names mouse/human (Ncor1, NCOR1 and Ncor2, NCOR2). Line 53: bind to their nuclear receptor partners THROUGH (not thorough).

Our apologies. We have corrected these errors and proofread the revised manuscript.
