# Supplementary material for: Specific ablation of the NCoR corepressor δ splice variant reveals alternative RNA splicing as a key regulator of hepatic metabolism
Source: PLoS One. 2020 Oct 26;15(10):e0241238. doi: 10.1371/journal.pone.0241238 (PMC7588069; doi:10.1371/journal.pone.0241238)
Supplement: Attachment — Submitted filename: Authors response 100220.docx [file pone.0241238.s007.docx]

Authors' response to reviewers' comments on PONE-D-20-17204-R1:

Again, we sincerely thank the reviewers and the editor for their efforts on our behalf. We believe that their suggestions have improved our manuscript. We hope that we have adequately answered their concern and that the revised manuscript is now acceptable for publication. We detail our response below:

**Reviewer #1 requested that we discuss whether the largely opposing effects we observe with the ablation of NCoRδ and NCoRω on steatosis and cholesterol metabolism can be related to the differences in affinity for various nuclear receptors between NCoRδ and NCoRω that are observed in vitro. Reviewer 1 also requested that we provide more detailed data and methods for the GST pulldown analysis of corepressor isoform-nuclear receptor interactions. :**

1. The authors have already stated the different partner interaction with the two different corepressor splice variants by GST-pull down experiments. Please discuss whether the changes in expression of sets of genes associated with hepatic steatosis and cholesterol metabolism exhibited largely opposite profiles in the NCoRδ-/- versus the NCoRω-/- livers were caused owing to different affinities of NCoRδ and NCoRω for many of nuclear receptors or other partners. Please provide the detailed method and results for GST-pull down in the manuscript.

We now discuss one possible mechanism by which the differences we observe in affinity between NCoRδ and NCoRω for LXRα could lead to changes in hepatic steatosis and cholesterol metabolism through changes in Srebf1 expression (lines 539-558). While this mechanism very likely contributes to the enhanced hepatic steatosis in the livers of the NCoRω-/- mice that is not observed in the livers of the NCoRδ-/- mice, it also seems very likely that other nuclear receptors and transcription factors, both within the liver and in other tissues, likely also contribute to the metabolic phenotypes we observe in the liver (lines 513-527).

Quantification data from the GST pulldown assays used to calculate the affinity ratios (lines 325-329) for NCoRδ:NCoRω in the text of manuscript are now provided in Table S3. A detailed method for the GST pulldown assays is also provided (lines 148-177).
